# Supplementary figures and images for: Chloroquine decreases cardiac fibrosis and improves cardiac function in a mouse model of Duchenne muscular dystrophy
Source: PLoS One. 2024 Jan 31;19(1):e0297083. doi: 10.1371/journal.pone.0297083 (PMC10830020; doi:10.1371/journal.pone.0297083)

Suppl Fig

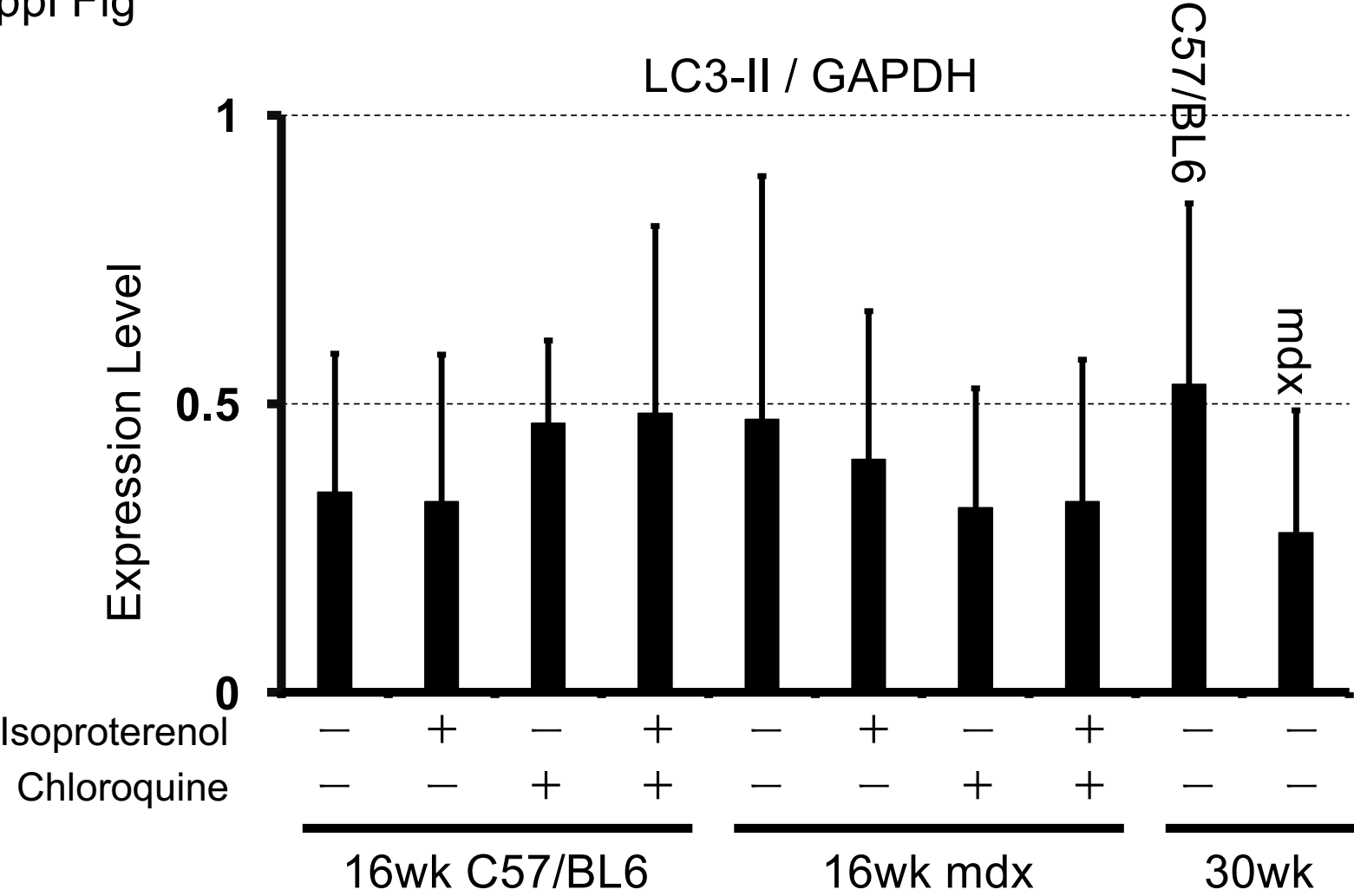

Supplement: S1 Fig — No significant difference between the expression ratio of each group. (PDF) [file pone.0297083.s001.pdf]
