# Supplementary material for: Chloroquine decreases cardiac fibrosis and improves cardiac function in a mouse model of Duchenne muscular dystrophy
Source: PLoS One. 2024 Jan 31;19(1):e0297083. doi: 10.1371/journal.pone.0297083 (PMC10830020; doi:10.1371/journal.pone.0297083)

1) LC3-I, LC3-II gel image

The expressions of LC3-I and LC3-II are indicated with arrows. Cut-out images of LC3-I and LC3-II bands are shown in Fig 3.

⟵ : LC3-I expression (17kDa)

⇠ : LC3-II expression (13kDa)

The image was captured with ChemiDoc XRS Plus (BIO-RAD).

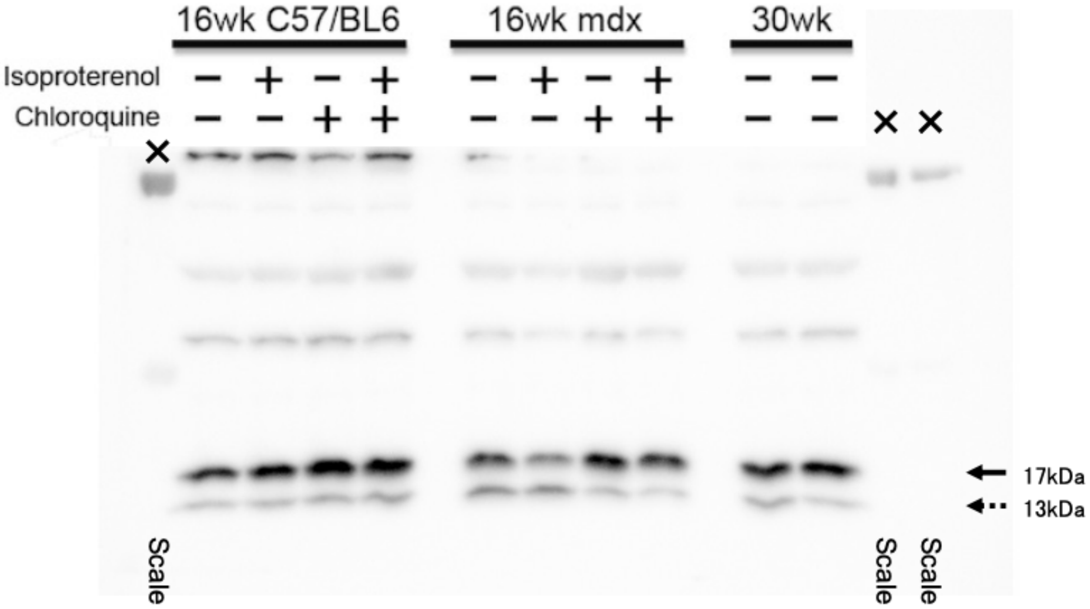

2) p62/SQSTM-1 gel image

The expression of p62/SQSTM-1 is indicated with an arrow. The cut-out image of p62/SQSTM-1 (62kDa) bands is shown in Fig 3.

The image was captured with ChemiDoc XRS Plus (BIO-RAD).

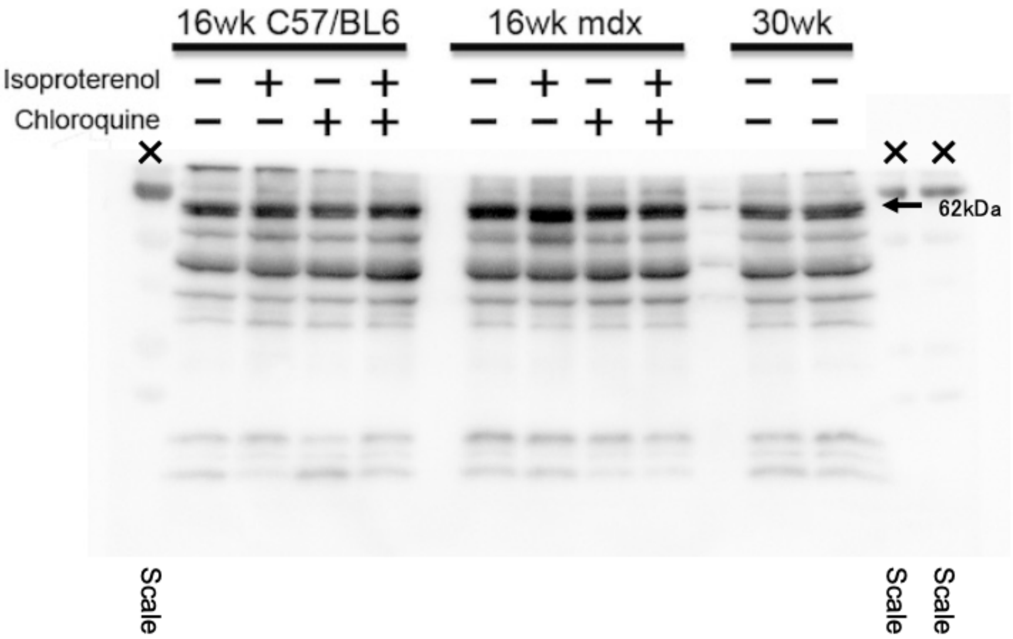

3) GAPDH gel image

The cut-out image of GAPDH bands (36kDa) is shown in Fig 3.

The image was captured with ChemiDoc XRS Plus (BIO-RAD).

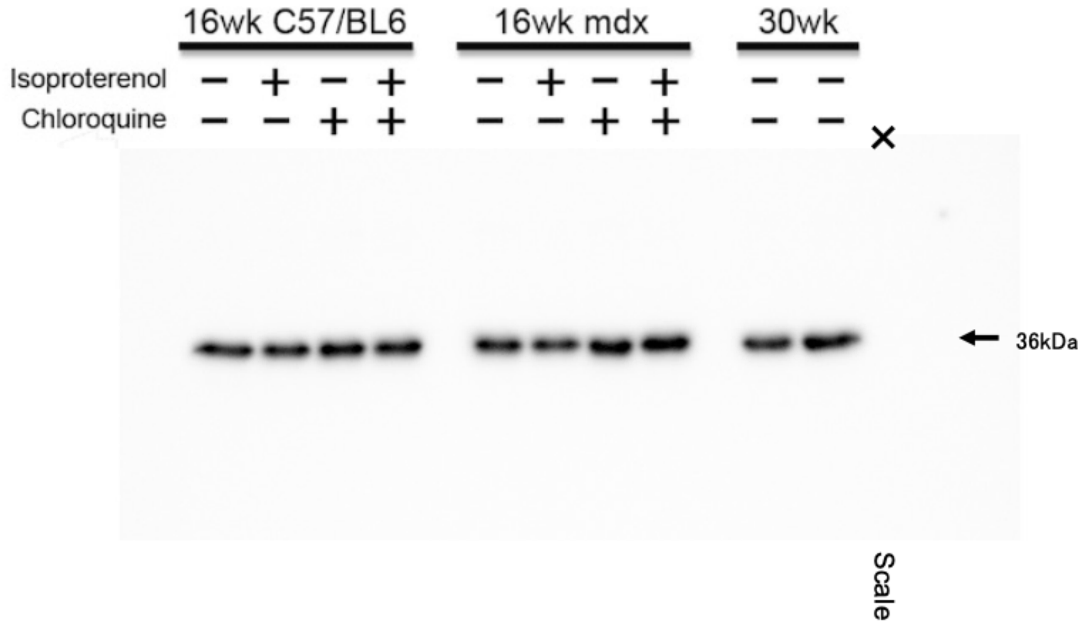

Supplement: S1 Raw images — 1) LC3-I, LC3-II gel image: The expressions of 17kDa LC3-I and 13kDa LC3-II were identified by Western blotting. Cut-out images of LC3-I and LC3-II bands are shown in Fig 3. 2) p62/SQSTM-1 gel image: The expressions of 62kDa p62/SQSTM-1 were detected by Western blotting. Cut-out images of p62/SQSTM-1 bands are shown in Fig 3. 3) GAPDH gel image: 36kDa GAPDH bands were detected. The cut-out image is shown in Fig 3. (PDF) [file pone.0297083.s002.pdf]
