# Supplementary material for: Chloroquine decreases cardiac fibrosis and improves cardiac function in a mouse model of Duchenne muscular dystrophy
Source: PLoS One. 2024 Jan 31;19(1):e0297083. doi: 10.1371/journal.pone.0297083 (PMC10830020; doi:10.1371/journal.pone.0297083)
Supplement: Attachment — Submitted filename: Respond to Reviewers.docx [file pone.0297083.s003.docx]

Thank you very much for all your comments, advice and questions.

All comments helped us to revise and improve our manuscript. I have written my answers to each question, and the corrected parts of the manuscript are noted in red color and underlined.

**Reviewer #1:**
The question asked in this manuscript is a worthy question for the DMD research, clinician, and patient community. As the authors point out cardiomyopathy is now the leading cause of fatality amongst these patients. The methodology is sound, and the writing is mostly clear and concise.
I have five major issues –

1) My largest issue is the small number of repetitions.
a. As far as I can see the authors never state the number of animals used. From the graphs I am guessing 3 or 4, not enough to make such large claims.

Thank you very much for asking.

For electron microscopy assessments, LC3 staining, and Western blotting, we presented the average of three experiments each. For the left ventricular cardiac fibrosis, we performed experiments with five mice in each group. Regarding the echocardiogram examination, we conducted the experiment with 5 mice in each group. This frequency and the number are described in the Statistics paragraph of Materials and Methods.

b. Although the LC3 staining of figure 2 looks impressive, I need to see a quantitative and statistical examination of the staining.
c. Ditto for the western blots, additional samples are required for statistics.

Thank you for pointing it out.

For Western blotting, we performed three experiments and additionally presented a representative figure. The quantitative expression levels of the presented figures are presented in Figure 3. According to the revision, the original Figure 2 split into two figures, Figure 2 and Figure 3.

d. The echo data statistically support the conclusions
i. although p values are not provided – necessary for echo data.

Thank you for your advice.

The *p*-value for the significant difference in the echocardiogram is missing, so I added it to the Figure Legend of Figure 5. *: *p*<0.05 has been also added to the end of the text for other Figure Legends, Figure 2 and Figure 4.

ii. additional animals from different litters are required to verify the conclusions.

As you pointed out, you are right. Although *mdx* mice are a sibling of C57/BL10 mice, they are also genetically close to C57/BL6 mice, so we used C57/BL6 mice because we thought there was a high possibility of obtaining similar data. Moreover, we used C57/BL6 because it took a very long time for our facility to purchase and obtain C57/BL10 mice.

2) The histochemistry figures are sub-standard.
a. The background staining is not uniform, so one cannot draw conclusions regarding red intensity.

Thank you for your advice. As you pointed out, it is true that the background of the staining is somewhat uneven, BZ-X-Analyzer software was used to automatically recognize the fibrotic areas and normal cardiomyocyte areas by observing the contrast difference between the fibrotic and normal areas. The area of the fibrosis was automatically calculated. The microscope we used this time was not a BZ-9000, but a BZ-X-710. We revised the "Histology and immunohistochemistry" section of Materials and Methods.

b. The horizontal bars in A, b, are likely artifacts and make any quantitation difficult.

As mentioned above, the fibrotic areas and normal areas are calculated using BZ-X-Analyzer software, which automatically recognizes the fibrotic areas, so there is no need for human intervention for the calculation.

c. Additional animals are required and maybe a different stain – Collagen, fibronectin... IF

As you pointed out, I performed Masson-Trichrome staining. It was clear that there were a lot of fibrotic areas in *mdx* mice left ventricle sections compared to WT mice, which tended to be suppressed by chloroquine. There were some parts that were difficult to determine the fibrotic area or not by this staining, so it could not be evaluated accurately. However, since a similar trend was observed, thus, the results of Picro-Sirius Res staining seem to be correct.

3) The authors should have done a cursory skeletal muscle including diaphragm, phenotyping to assess the impact of chloroquine on the other DMD affected muscles. As they themselves point out in the discussion (lines 221-2 for example).

That's right. However, since cardiac fibrosis begins much later than skeletal muscle fibrosis, it is difficult to evaluate skeletal muscle at the time of cardiac evaluation using this experimental protocol. Actually, there are many papers that show that autophagy is reduced in skeletal muscle, so we discussed skeletal muscle autophagy by using previous papers in the Discussion section.

4) The text is unclear on autophagy in mdx skeletal muscles. In the introduction autophagy is said to be upregulated in mdx skeletal muscles (lines 28-29). In the conclusion autophagy is said to be decreased in DMD patients and mdx mice (lines 216-7). Then later in the conclusion autophagy is decreased (lines 221-2).

Thank you for your advice.

Previous reports have consistently shown that autophagy is reduced in skeletal muscle. The original submitted manuscript in lines 28-30 does not state that autophagy is increased in skeletal muscle, but it is certainly an ambiguous expression to understand. So, we revised the sentence to "Because decreased autophagy has been reported to contribute to skeletal muscle degeneration, therapies targeting autophagy are expected to improve skeletal muscle hypofunction.”

5) In the cited work by the Selsby team they found DECREASED autophagy in mdx cardiac tissue. In previous work by Bibee KP 2014 and Pal R 2014, increasing autophagy aided the mdx cardiac muscles, in contrast to your autophagy inhibition. Additional paragraphs are required to clarify these discrepancies.

Selby's research results showed that p62 expression in *mdx* mice remained unchanged, LC3-II increased, and LC3-I decreased. Thus, he concluded that the influence of autophagy in the cardiomyocytes is unknown. Bibee KP and Pal R concluded that the therapeutic strategy for DMD patients was to rescue the reduced autophagy in cardiomyocytes. Our Western blotting and LC3 dots results showed isoproterenol-induced autophagy in *mdx* mice cardiomyocytes, but there is little change in p62, and the LC3-II/LC3-I ratio decreases upon chloroquine administration. Considering this, it seems that there is still insufficient evidence to consider that autophagy is the definitive cause of cardiomyocyte changes in *mdx* mice. Therefore, we believe that mechanisms other than autophagy should be evaluated in the future. We also think that the effect of chloroquine on autophagy should be re-evaluated. Based on the above, we have changed some sentences of the abstract and discussion, and changed the title to “Chloroquine decreases cardiac fibrosis and improves cardiac function in a mouse model of Duchenne muscular dystrophy.”

Minor issues –
1) The statistics section is unclear. “All expts were performed at least three times” tells the reader very little. We need to know number of mice per group and/or per expt. Do you mean three echoes per mouse? Three histologic slides per group, or per animal?

Thank you for your advice. As mentioned above, the number of experiments and the number of mice used are described in the statistics paragraph of Materials and Methods.

2) What is the comparison of P63 at baseline between the wt and mdx mice?

Considering the possibility that autophagy in *mdx* mice is enhanced even in an untreated state, we performed a baseline evaluation. The results showed no obvious significant difference.

3) Line 217 needs a reference, even if it is the same group as the following sentence.

Thank you for your advice. I have also inserted a reference number in the previous sentence.

4) I am bothered that you are really assessing the benefits of chloroquine in mdx mice that have been given isoproterenol. To state that chloroquine will benefit cardiac function in DMD patients is a bit of a stretch.

That's true, but there may be some influence on autophagy. I removed the word “autophagy” from the title and changed it to “Chloroquine decreases cardiac fibrosis and improves cardiac function in a mouse model of Duchenne muscular dystrophy.” Some sentences in the Results and the Discussion sections associated with this have also been changed.

**Reviewer #2:**

1. The studies referenced on page 12, line 154 were in myoblasts isolated from mice and should be references with caution, as autophagic flux can be vastly different between myoblast obtained from skeletal muscle to adult skeletal muscle to heart. In addition, these studies activated P2RX7 to induced autophagy in dystrophic myoblasts. The finding that LC3-I expression was stronger than LC3-II in mdx hearts under non-stressed conditions would suggest that in the absence of isoproterenol mdx hearts show an inhibition of autophagy, which is then inhibited further upon isoproterenol stimulation. CQ inhibits autophagy at the late stages and therefore promotes accumulation of markers such as LC3 and p62, thus an increased LC3 is indicative of inhibition of autophagy, not increased autophagy. It is not clear how CQ would decrease these markers. The failure of CQ to alter the markers of autophagy in WT and mdx suggests that the concentration of CQ was not sufficient to modify autophagy. Authors would need to show that CQ promotes a strong accumulation of LC3 and p62 in both wild-type and mdx tissues in order to conclude that CQ has any effect in these studies.

Thank you for asking. The concentration of chloroquine used in this study is determined based on previous papers. Although it was correctly evaluated that chloroquine suppresses cardiac fibrosis, considering our results, there is a possibility that the inhibition of autophagy by chloroquine was mild in our experiments. Therefore, we added some sentences in the Discussion section that mentioned the possibility that autophagy might not be completely suppressed due to the administration amount of chloroquine.

2. To this reviewer the changes observed in figure 2 are modest to no change. Quantification of autophagy markers is needed to draw conclusions regarding the status of autophagy. Furthermore, the authors need to show and quantify more than a single sample from each group in order to properly evaluate the reported outcomes. Clearly state in the methods and/or figure legends the number of mice used for each group for each experiment.

The number of mice was 5 each for echocardiography and fibrosis evaluation, and 3 mice each for Western blotting and LC3 experiments. These are described in the Statistics paragraph of the Materials and Methods section. Regarding the quantification of Western blotting, the signal intensity was quantified using BIO-RAD Image Lab 6.0 software, and the ratio with GAPDH was determined. Regarding dots, we counted three locations for 0.01mm2 in the left ventricle cross-section at the papillary muscle level. These were added to the Materials and Methods section, and their quantified graphs were presented in Figures 2 and 3.

3. The authors state that their findings indicate that cardiac stress induces cardiac regeneration in mdx mice is unexpected. Cardiac regeneration is a hot topic in the field and the consensus is that adult hearts do not regenerate. It is not clear from the presented data how the authors came to the conclusion that isoproterenol stress promotes regeneration in the mdx hearts since there was increased fibrotic scaring. In order to draw such conclusions the authors would need to assess/measure markers of cardiac regeneration.

Myocardial regeneration is an interesting research topic. However, although changes in autophagy were described, regeneration was not mentioned in this paper, and the regeneration has not been evaluated experimentally.

4. Page 17 lines 228-229 the referenced paper from Spaulding et al is a bit misleading, as the title of the paper states enhanced autophagy in dystrophic heart; however, the conclusions stated by the authors are that autophagy is not impaired in the hearts and that disease progression is independent of autophagic dysfunction. In the current work the authors find no change in autophagy in mdx hearts compared to WT, thus in this context they have confirmed the finding of Spaulding et al. However, neither study show that under non-stressed condition autophagy is enhanced in mdx hearts. The conclusions drawn from this study that autophagy inhibition (by CQ) inhibits autophagy, leading to improved cardiac function are not supported by the data presented. CQ did not inhibit autophagy (see specific comments regarding this above). It is interesting that CQ did improve stress induced function and at the same time decreased fibrosis. In addition to inhibition of autophagy CQ is known to modify the immune response, including Toll like receptors and the cGAS-Sting pathways which promotes fibrosis. This raises the possibility that in the current studies CQ is inhibiting fibrosis through these pathways in non cardiomyocytes rather than through inhibition of cardiomyocyte autophagy.

Our data showed that autophagy may affect cardiac change in the *mdx* mice. In fact, autophagy was enhanced by isoproterenol administration, and cardiomyopathy was improved by chloroquine treatment. Based on the results of p62 expression and LC3, besides autophagy, we also believe that the immune response and anti-inflammation effects of chloroquine possibly influenced the results to some extent. Therefore, we have added these considerations to the Discussion section.

**Reviewer #3:**

1. Chloroquine is well characterized to inhibit autophagosome fusion to lysosome, which leads to autophagic flux impairment, evident by the accumulation of LC3. It is thus very surprising and unexpected that the chloroquine treatment seems to reduce LC3 as shown by immunohistochemistry and consistent with the lack of LC3 increase by protein blot. There is also no increase in p62, suggesting that chloroquine in this case is not reducing autophagy by blocking autophagic flux. Since this effect of chloroquine has never been reported in literature, what is the authors’ hypothesis on how chloroquine is reducing autophagy in this case? Is it possible that the protective effect seen here is unrelated to the role of chloroquine to inhibit autophagosome-lysosome fusion? Much more detailed investigation is likely needed to support this controversial claim that chloroquine reduces autophagy, or more specifically autophagosomes.

Thank you for your advice.

Our data suggest that autophagy may be involved in the cardiomyopathy of *mdx* mice, and in fact, autophagy was enhanced by isoproterenol and cardiac fibrosis was shown to be improved by treatment of chloroquine. However, from p62 and LC3 expression results, it is needed to evaluate the dose-dependent effect of chloroquine administrated to mice. It was thought that further experiments and consideration should be done. We also believe that the effects of chloroquine on immune responses and controlling inflammation may have influenced our results. Therefore, I added these discussion sentences in the Discussion section.

2. In Fig. 2A (electron microscopy panel), the figure legend states that the scale bar is 100 um, which seems unlikely. Is that correct? Also please specify which cohort of mice was this image taken from. Thirdly, please also include a more magnified image of the autophagosome.

Thank you for your advice. The scale comment was incorrect. The scale bar is 100nm. Tissues shown in Figure 2A are cardiomyocytes from *mdx* mice administered isoproterenol in Figure Legends.

3. In Fig. 2B, please show quantification of the LC3 puncta. Also please specify in the figure legend and detail in the method how many mice were examined, how many sections, and how the LC3 puncta were determined and counted.

Thanks for your questions.

Quantification of LC3-positive dots was performed by automatic counting. The method was to automatically count three 0.01mm2 areas in each left ventricle section in each group using BZ-X-Analyzer software and Photoshop software. Figures 2 and 3 have been revised accordingly. The method is described in Materials and Methods section.

4. In Fig. 2C, please show quantification of the protein blot data. Also please specify how many mice were examined from each group and how many technical replicates were performed.

Three mice were used in each Western blotting experiment. The signal intensity was quantified using BIO-RAD Image Lab 6.0 software, and the ratio with GAPDH was determined. Regarding dots, three locations were counted for each 0.01 mm^2^. I added the graph and revised Figures 2 and 3.

5. To confirm whether autophagic flux in increased or inhibited, please also include protein blots from each treatment group where autophagy is blocked before sacrifice (for instance inject mice with autophagy blocker bafilomycin 2 hours prior to sacrifice) for comparison. The data presented is not sufficient to support the authors’ claims that autophagy is activated in mdx mice injected with isoproterenol, and would further strengthen the mechanism of how chloroquine can reduce LC3.

Thank you for your advice. I think this is definitely a necessary experiment. However, there are many unknowns, such as the dosage and administration timing of bafilomycin. We tried to forcibly administrate bafilomycin orally. When mice were administered the drug, they often vomited, and the experiment was done unsuccessfully. We plan to reconsider the methodology and conduct this as a future experiment.

6. In Fig. 3A please use arrow or magnified panels to indicate the areas of fibrosis. The figure legend states that the scale bar is 100 um which seems unlikely. Please correct. Also please elaborate in the Methods on how fibrosis area is quantified (for instance how were the threshold determined by the software etc.) and how many animals and how many sections were analyzed.

Thank you very much for your advice.

The scale bar was actually 500 μm. For the evaluation of fibrosis, five mice were used in each group. All areas of the short-axis left ventricle cross-section slices at the level of the left ventricle papillary muscle are counted (calculated). We used BZ-X-Analyzer software to automatically recognize fibrotic and normal areas, and calculated fibrotic area ratio per the short-axis left ventricle cross-section slices. The microscope we used was not a BZ-9000, but a BZ-X-710. We have revised the Materials and Methods section.

7. How was chloroquine encapsulated for subcutaneous delivery? Please specify in Materials and Methods.

Chloroquine was dissolved at 75 mg in 250 mL of clean water and given to mice using an automatic water dispenser. It is not encapsulated, and the method was written incorrectly. We have corrected the description regarding this. We checked the amount of water loss every other day, and it was the same in all mouse groups.

8. The authors stated that “No mice treated with chloroquine exhibited side effects during the study”. Please specify what side effects were measured, were there any effects on skeletal muscle, and what were the results.

Activity, appetite, and diarrhea were checked and none of these symptoms was noted. Check items have been added to the Materials and Methods section. Although the details of the effect on skeletal muscle are unknown, there was no significant difference in activity changes, and we believe that there is no obvious effect on skeletal muscle at least the chloroquine amount we administrated to the mice.

9. Please correct typos throughout the manuscript.

Thank you very much.
