# Supplementary material for: Chloroquine decreases cardiac fibrosis and improves cardiac function in a mouse model of Duchenne muscular dystrophy
Source: PLoS One. 2024 Jan 31;19(1):e0297083. doi: 10.1371/journal.pone.0297083 (PMC10830020; doi:10.1371/journal.pone.0297083)
Supplement: Attachment — Submitted filename: Response to Reviewers in 2nd revise-20231206.docx [file pone.0297083.s004.docx]

Thank you very much for all your comments, advice, and questions.

All comments helped us to revise and improve our manuscript. I have written my answers to each question, and the corrected parts of the manuscript are noted in red color and underlined. In addition, we focused on the cardiac fibrosis phenotype rather than the autophagy mechanism in our revised manuscript. Thus, a lot of sentences in all sections were revised.

Reviewer #1: (No Response)

Reviewer #2:

1) While the authors were responsive to several minor concerns raised, they were not responsive to the main concerns that provide uncertainty on the conclusions drawn from their data. The concern of the interpretation of the CQ results was raised by more than one reviewer and yet the authors have not provided insight into how the authors think CQ is reducing autophagy. To reiterate the concern, blocking of autophagy using CQ is usually characterized by accumulation of LC3 and p62; however, these markers change in the opposite direction. So, how might CQ work differently in this study?

（Answer）　Based on our data from this study, we could not conclude that the improvement of cardiac fibrosis was a result of autophagy. However, the histological results showed that administration of isoproterenol caused more fibrosis in the cardiomyocytes of *mdx* mice, and chloroquine probably suppressed the fibrosis of myocardial cells by suppressing stress (isoproterenol)-induced inflammation rather than autophagy. According to this idea, we revised sentences in the “Abstract” and the “Discussion” sections.

2) In the conclusion section of the abstract the authors state the mechanism of the effect of CQ may involve immune control and anti-inflammatory effects. To state this as a conclusion leads a reader to anticipate data supporting the conclusion; however, no data is presented. While these pathways may be a possibility, and warrant a discussion in the discussion section, the statement is not a conclusion of the study.

(Answer) Thank you very much. Logically, your comment is correct. For revising our manuscript, we tried to confirm whether isoproterenol-induced cardiac inflammation and chloroquine suppressed the inflammation assessed by the expression of NFK-B in the western blotting method. As shown in the graphs presented below, the expression levels showed wide variation. Therefore, the conclusion that chloroquine suppresses the isoproterenol-induced inflammation could not be theoretically drawn from the assessment by western blotting expressions of NFK-B. Possibly, we have to elucidate the inflammatory mechanisms by using other inflammatory pathways. However, considering the phenomena of our experiments and previous reports, I think that chloroquine may suppress inflammation in cardiomyocytes. The sentences in the conclusion in the “Discussion” section have been changed.

3) Figure 2A: a comparison between wt and mdx is needed for the electron microscopy. Quantification of autophagosomes between the 2 genotypes from the electron micrographs is also needed.

(Answer). Thank you for your comment. Autophagosomes were rarely found in cardiomyocytes of control mice cardiomyocytes and were difficult to quantify.

4) Figure 2B: the data does not support the statement that “abundant LC3 positive dots were present in mdx mouse cardiomyocytes”. Both the images and the quantification does not show increased LC3 positive structures in the mdx compared to wt. The quality of the images in panel B can be improved in order to assess localization of the LC3 puncta. It is difficult to know where the cardiomyocyte boundary is and whether the LC3 are within the myocycte or in the interstitial space. This last point may also provide important information as to whether the effects observed in these studies are on cardiomyocytes or other infiltrating cells as I previously raised.
Line 168-169: Authors state “When an autophagy inhibitor, chloroquine, was administrated to mdx mice, the ratio of LC3-I and LC3-II expression levels did not change even after the administration of isoproterenol.” The interpretation of this sentence is that 1. CQ does not change the LC3II/Lc3I in mdx mice. This is not supported by the date presented, although there are no stats provided the trend is that the level is much lower in mdx+CQ compared to mdx. 2. Iso+CQ is not different than mdx, again not supported by the data. The reporting of the data needs to more clearly and accurately describe what the data show. Also, while the authors now indicate that 3 mice were evaluated for these studies they did not provide the blots of all the mice (only show the same data in the supplement as the main figure) and do not show a quantification of all 3 in the bar plot, as one would expect to see some indication of variance in the data. Is the data presented in panels B and C and average of the 3 mice or just that of the one mouse shown in panel A? The authors need to show and quantify more than a single sample from each group in order to properly evaluate the reported outcomes.

(Answer) For dot data images, high-resolution data will be submitted again. In addition, the quantified western blotting data in Figure 2 are presented as the average and standard deviation of data obtained from three individual mice in each group and three separate experiments. The number of autophagosome dots was small both in cardiomyocytes of *mdx* and WT mice. According to the data, we revised sentences in the “Abstract”, “Materials and Methods”, “Results”, and “Discussion” sections.

5) Line 194: The authors state “These findings indicated that cardiac stress significantly induced cardiac regeneration in mdx mice.” This conclusion is not supported by the data. There is no evidence of increased cardiac regeneration. This should be removed.

(Answer) Thank you for this comment. We removed the sentence from our manuscript.

6) There are a number of misspellings and misidentifications (e.g. use of p63 instead of p62 and indicating LC3I/LC3II expression when LC3II/LC3I is shown).

(Answer) Thank you very much. We checked and corrected all according to your comments.

Reviewer #3: I appreciate the authors’ response to the review. I have the following requests and questions:

7) In the now Figure 3, please quantify LC3-II to GAPDH ratio as well. LC3-II is a well-accepted marker of autophagosome and in the interpretation of autophagic flux.

(Answer). Thank you for your comment. We added the data of LC3-II/GAPDH expression by western blotting as a supplemental figure. The level of the expression did not differ significantly in each group.

8) In the now Figure 3 quantification panels, please also plot the standard deviations and perform statistical testing (ANOVA with post-hoc test) to indicate whether there is statistical difference in LC3-II, LC3-II/I and p62 protein expression among the groups.
Please revise the description of the Figure 3 results (Lines 160-182) depending on the results of the statistics. It is a bit confusing especially without the statistical testing.

(Answer) You're right. I have corrected the sentence.

9) In Lines 180-182, the authors stated that “These findings indicated that autophagy occurred constitutively in mdx mouse cardiomyocytes and that mdx mouse cardiomyocytes were more sensitive to cardiac stress than WT mouse cardiomyocytes.” Please discuss this instead in the Discussion section, and clarify what the authors mean by autophagy occurring constitutively, and how this leads to the conclusion that there is a difference in the sensitivity to cardiac stress. Secondly, since I assume the western blot is performed on heart tissue lysates please refrain from making the conclusion on cardiomyocytes since other cardiac cell types are present.

(Answer) Thank you for your comment. We believe that constitutively observed autophagy may induce a long-term decline in cardiomyocyte function. However, we could not reach a piece of clear evidence to prove the relationship between cardiomyopathy and autophagy. I have mentioned this in the “Discussion” section.

10) Please correct “p63” to “p62”.

(Answer) Thank you very much. I have corrected the words in the manuscript.

11) It is interesting that LC3 puncta are significantly increased in mdx mice treated with isoproterenol compared to mdx mice without isoproterenol in Figure 2, but both LC3-I and LC3-II seemed to be reduced in the western blot data. What is the explanation? Please elaborate in the Discussion section.

(Answer) Thank you for your question. It is a very important matter. Although the increased number of autophagosomes is probably the result of induction of the autophagy pathway, it is thought that what was suppressed by chloroquine was not autophagy but inflammation of cardiomyocytes. Therefore, I think that autophagy does not cause cardiomyocyte fibrosis. Although within the scope of discussion, isoproterenol administration induces both autophagy and inflammatory pathways, suggesting that the inflammatory pathway may contribute to cardiomyocyte fibrosis. I wrote about this consideration in the “Discussion” section.
